# Breast Volume Measurement by Recycling the Data Obtained From 2 Routine Modalities, Mammography and Magnetic Resonance Imaging

**Published:** 2017-12-20

**Authors:** Shizu Itsukage, Yoshihiro Sowa, Mariko Goto, Tetsuya Taguchi, Toshiaki Numajiri

**Affiliations:** ^a^Departments of Plastic and Reconstructive Surgery; ^b^Radiology; ^c^Endocrine and Breast Surgery, Kyoto Prefectural University of Medicine, Graduate School of Medical Sciences, Kyoto, Japan

**Keywords:** breast volume, magnetic resonance imaging, breast reconstruction, mastectomy, breast cancer

## Abstract

**Objective:** Preoperative prediction of breast volume is important in the planning of breast reconstructive surgery. In this study, we prospectively estimated the accuracy of measurement of breast volume using data from 2 routine modalities, mammography and magnetic resonance imaging, by comparison with volumes of mastectomy specimens. **Methods:** The subjects were 22 patients (24 breasts) who were scheduled to undergo total mastectomy for breast cancer. Preoperatively, magnetic resonance imaging volume measurement was performed using a medical imaging system and the mammographic volume was calculated using a previously proposed formula. Volumes of mastectomy specimens were measured intraoperatively using a method based on Archimedes’ principle and Newton's third law. **Results:** The average breast volumes measured on magnetic resonance imaging and mammography were 318.47 ± 199.4 mL and 325.26 ± 217.36 mL, respectively. The correlation coefficients with mastectomy specimen volumes were 0.982 for magnetic resonance imaging and 0.911 for mammography. **Conclusions:** Breast volume measurement using magnetic resonance imaging was highly accurate but requires data analysis software. In contrast, breast volume measurement with mammography requires only a simple formula and is sufficiently accurate, although the accuracy was lower than that obtained with magnetic resonance imaging. These results indicate that mammography could be an alternative modality for breast volume measurement as a substitute for magnetic resonance imaging.

Breast volume is important for planning of breast reconstruction after mastectomy, but breast volume measurements are not widely performed because there is no standard method. The ideal measurement would be fast, simple, and, above all, accurate, with the use of a procedure that does not require extra time, cost, and discomfort for patients. From this perspective, data from routine modalities such as mammography (MMG) would be a good option for breast volume measurement. Breast magnetic resonance imaging (MRI) has also become a common modality for diagnosis and tumor evaluation in many countries because of the accuracy of this method for cancer staging and tumor extent.^1^^-^3 Breast volume can be calculated through reconstruction of 3-dimensional (3D) images from MRI, using specific analysis software. The purpose of this study was to calculate breast volumes prospectively using data obtained from MMG and MRI and to estimate the accuracy of these measurements by comparing the results with volumes of mastectomy specimens.

## PATIENTS AND METHODS

### Subjects

The subjects were 22 patients (24 breasts) who had been diagnosed with breast cancer and were scheduled for total mastectomy from September 2013 to March 2014 at our hospital. All had undergone routine preoperative MMG for diagnosis and breast MRI for evaluation of the extent of the tumor. After detailed examinations for treatment planning, mastectomy was performed. The study was approved by the ethics committee of our hospital (Kyoto Prefectural University of Medicine).

### Breast volume measurement using MRI

**Figure 1:**
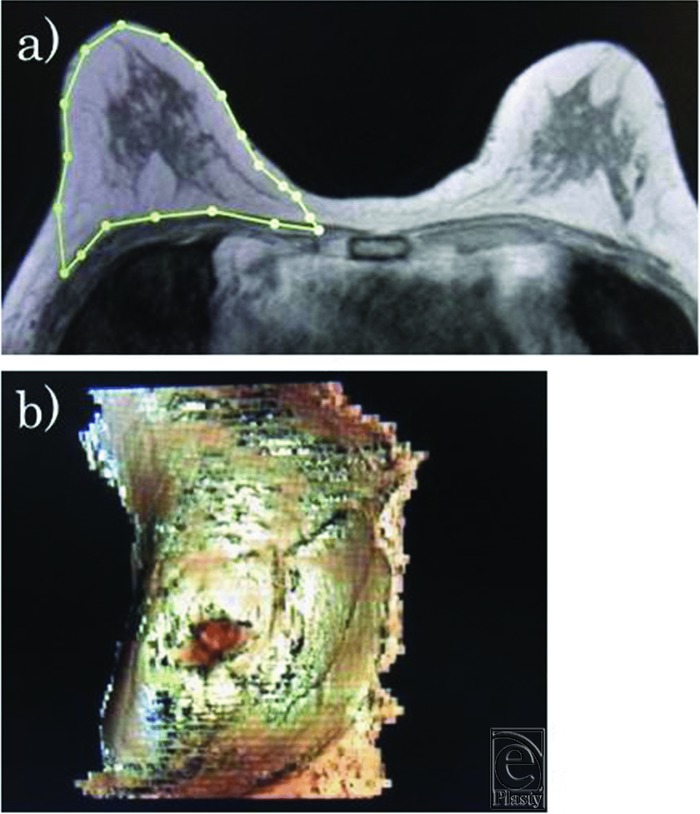
Breast volume measurement by magnetic resonance imaging. (a) Outline of breast tissue. (b) Reconstructed 3-dimensional image and calculation of the breast volume.

Breast 3.0-T MRI scans were conducted in a prone position. A medical imaging system (AQI Viewer, Terarecon Inc, Foster City, CA) was used for MRI volume measurement. An outline of the mammary gland was plotted manually using about 15 slices per breast. The area of interest was integrated into a 3D image by the imaging system, and the breast volume was calculated using a volumetric tool (Fig 1). Measurements were performed using axial and sagittal views, and the average was taken to be the MRI breast volume (*V*_MRI_). The time required for each measurement was recorded.

### Mammographic volume measurement

**Figure 2:**
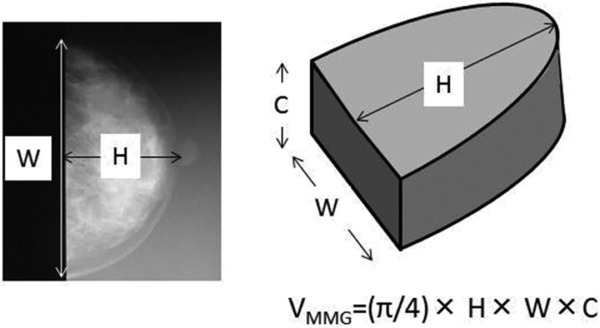
Breast volume measurement by mammography. The breast volume is calculated using a formula reported by Kalbhen et al.^4^ *V*_MMG_ indicates breast volume measured using mammography; H, breast height (cm); W, breast width (cm); and C, compression thickness (cm).

Breast volume based on MMG data was calculated using the formula proposed by Kalbhen et al^4^: *V*_MMG_ = (π/4) × Breast height × Breast width × Compression thickness in craniocaudal MMG. This calculation is based on the assumption that the shape of the compressed breast in the craniocaudal projection can be regarded as a half-elliptical cylinder (Fig 2).

### Measurement of mastectomy specimen volume

The method proposed by Fujiwara et al^5^ that incorporates Archimedes’ principle and Newton's third law was used to measure the mastectomy specimen volume. Briefly, a container filled with saline is put on a weight scale. After zero-point adjustment, the mastectomy specimen is immersed under the saline in the container. The value on the scale is equal to the buoyancy the specimen provides and the weight of saline displaced by the specimen. The specimen volume (*V*_sp_) can be obtained by dividing the value on the scale by the density of saline (1.006 g/mL). This technique gives a highly accurate specimen volume.

### Accuracy of breast volume measurement

Differences in mean *V*_MRI_, *V*_MMG_, and *V*_sp_ were evaluated, and linear regression models were used to obtain correlation coefficients. *P* < .05 was considered to be significant. Statistical analysis was performed using InStat ver. 3.0 (GraphPad, New York, NY).

## RESULTS

**Figure 3:**
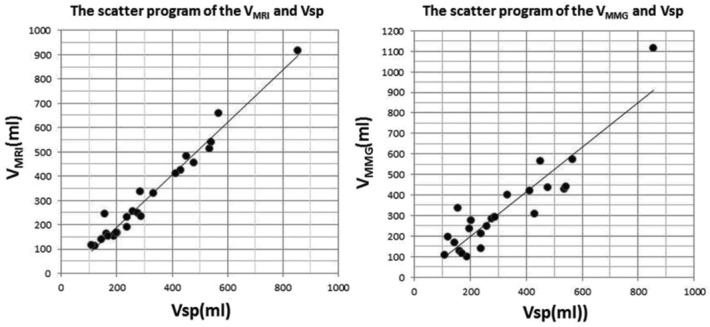
Scatter plot of *V*_MRI_ and *V*_sp_. The correlation coefficient (*r*) was 0.982 (*P* < .05). *V*_MRI_ indicates breast volume measured using magnetic resonance imaging; *V*_sp_, volume of mastectomy specimen.

The mean age of the patients was 53.34 years (range, 31-84 years). The mean value of *V*_MMG_ and *V*_MRI_ was 325.26 ± 217.36 mL and 318.47 ± 199.4 mL, respectively, and that of *V*_sp_ measured intraoperatively was 318.86 ± 181.82 mL. The mean errors were 64.98 ± 62.71 mL for *V*_MMG_ and 27.94 ± 26.97 mL for *V*_MRI_. Scatter plots of *V*_MRI_ and *V*_MMG_ with *V*_sp_ are shown in Figure 3 respectively. Both methods showed linear associations with *V*_sp_, with a slightly higher Pearson correlation coefficient for *V*_MRI_ (0.982; 95% confidence interval [CI], 0.960-0.992; *P* < .05) than for *V*_MMG_ (0.911; 95% CI, 0.802-0.961; *P* < .05). The average time required for measurement using MRI and MMG was 160 and 115 seconds, respectively.

## DISCUSSION

Accurate and objective measurement of breast volume is helpful for choosing an appropriate breast implant and determining the transplanted volume of autologous tissue in breast reconstruction surgery. Several methods for measuring breast volume have been described, but most require further examination, time, cost, and an extra burden on patients. MMG has been commonly used for breast cancer diagnosis for many years, and in the past 2 decades, MRI has been increasingly used for diagnosis and evaluation of tumor extent in breast cancer, as recommended by various national standards and guidelines.^1^^-^3 MRI screening for high-risk patients is recommended in the guidelines updated in 2008 by the American Cancer Society because MRI is more sensitive than MMG. More recently, MRI has been recommended as the first choice for estimation of tumor extent in breast cancer, especially in Japan.

In the current study, we evaluated the accuracy of breast volume measurement using MMG and MRI because use of these routine modalities reduces the financial, physical, and mental burden on patients and the data can be obtained easily in a clinical setting. Breast MRI is performed in a prone position, with the breast outlined to grasp the extent and characteristics of the tumor. The contrast of breast tissue and skin is depicted clearly, which allows for simple plotting of the outline of breast tissue. Our results showed a strong linear association between breast volumes of surgical specimens and those measured by 3D MRI. The 3D MRI model of the breast can be easily developed, and the breast volume calculations are simple and rapid and based on routine preoperative data. The time taken to calculate the breast volume with MRI data was only 160 seconds, which indicates that the method is simple for both patients and medical staff.

We added 2 refinements to previous measurements of breast volume using MRI.^6^^,^^7^ First, our study is prospective. Second, the surgical specimen volume was measured with a more accurate method in accordance with Archimedes’ principle and Newton's third law.^5^ This is in contrast to methods in which the surgical specimen volume is obtained by simply dividing the weight by a constant density of breast tissue^6^ or by assuming an approximate weight.^7^ The proportion of breast glandular tissue and fatty tissue is likely to vary among individuals. Therefore, our method provides stronger evidence that breast volume measurement with 3D MRI is accurate. Nevertheless, MRI has a disadvantage of requiring specific analysis software.

Our findings showed that breast volume measurement with MMG is accurate despite the lower accuracy than MRI. We used a relatively simple formula based on a half-elliptical cylinder model among several proposed formulas.^4^^,^^8^^,^^9^ Given that the breast volume calculation from MMG is simple, is not highly variable among individuals, and can be performed without extra time, cost, and discomfort for patients, MMG is a candidate modality to provide information for planning reconstructive breast surgery when MRI or volume analysis software is unavailable.

To the best of our knowledge, this is the first study of breast volume measurement using data from routine modalities of MMG and MRI. Estimating breast volume using a combination of these two modalities may facilitate more accurate and simple breast reconstruction.
